# Rising Carbon Dioxide and Global Nutrition: Evidence and Action Needed

**DOI:** 10.3390/plants11071000

**Published:** 2022-04-06

**Authors:** Lewis H. Ziska

**Affiliations:** Mailman School of Public Health, Columbia University, New York, NY 10032, USA; lhz2103@cumc.columbia.edu

**Keywords:** CO_2_, nutrition, protein, food web, iron, zinc

## Abstract

While the role of CO_2_ as a greenhouse gas in the context of global warming is widely acknowledged, additional data from multiple sources is demonstrating that rising CO_2_ of and by itself will have a tremendous effect on plant biology. This effect is widely recognized for its role in stimulating photosynthesis and growth for multiple plant species, including crops. However, CO_2_ is also likely to alter plant chemistry in ways that will denigrate plant nutrition. That role is also of tremendous importance, not only from a human health viewpoint, but also from a global food–web perspective. Here, the goal is to review the current evidence, propose potential mechanistic explanations, provide an overview of critical unknowns and to elucidate a series of next steps that can address what is, overall, a critical but unappreciated aspect of anthropogenic climate change.

## 1. Introduction

The impact of increasing anthropogenic global-warming gases in the context of physical disturbance, i.e., changes in temperature, rainfall, extreme storms and sea level rise is widely recognized by the public at large. Less recognized is the direct effect of a primary greenhouse gas and carbon dioxide (CO_2_) on plant biology. Yet, there are literally hundreds of studies showing that recent and projected increases in carbon dioxide concentration [CO_2_] can stimulate photosynthesis and the growth and phenology of approximately 90% of all known plant species, i.e., those who rely solely on the C_3_ photosynthetic pathway. As plants are autotrophs, and the sole suppliers of energy and elements to all living things, it is not an exaggeration to say that rising [CO_2_] will, by directly altering plant biology, influence all life on earth.

Should this influence then be seen as uniformly beneficial, a “green is good” counterweight to the physical disruptions associated with anthropogenic climate change? Caution should be exercised in such an interpretation, as it is also clear that [CO_2_] will have non-uniform benefits for plants, communities and ecosystems. For example, there is clear evidence that at the agricultural level, weeds can respond more than crops to [CO_2_], with consequences for crop production. For other biomes, there are reports that increasing [CO_2_] may favor the growth of invasive plants over native species [1], or vines over trees, [2] with subsequent disruptions to ecosystem function and biodiversity.

However, it is the direct effect of rising [CO_2_] on plant chemistry and stoichiometry that represents a continuing threat to the nutritional integrity of both human and animal systems. The earliest acknowledgement of [CO_2_] on nutritional aspects are by Sionit [3,4], reporting that leaves growing in elevated [CO_2_] had higher carbohydrate levels, higher C:N ratios and lower leaf nitrogen. These observations have gained credence over time, including multiple meta-analyses [5,6,7,8,9,10]. Currently, there is extensive evidence from multiple studies and meta-analyses that increasing [CO_2_] will reduce protein and mineral concentrations from a wide-variety of plant-based food sources, with substantial global consequences for human and animal nutrition. The basis for this reduction, an assessment of the potential impacts for human nutrition and for global food webs, critical unknowns and crucial next steps are assessed in the current review in the hope that attention can be paid to this overlooked and pervasive aspect of anthropogenic climate change.

## 2. Evidence and Mechanism

Crop breeding has not been static in the last 100+ years. Such efforts in conjunction with modern management techniques (fertilizer, pesticides) etc., reflect a hybridization of increased yield for numerous fruits and vegetables. It is reasonable to assume that modern crops that grow larger and faster may not acquire nutrients in proportion and are likely to decline in nutritional concentration. Jarrell and Beverly [11] provide an overview of a “dilution effect,” whereby yield-enhancing methods, such as fertilization and irrigation could decrease nutrient concentration. An evaluation of 43 foods (primarily vegetables) by Davis et al. [12] indicated statistical declines for protein, Ca, P, Fe riboflavin and ascorbic acid from 1950 to 1999, suggesting that cultivar selection for larger produce may represent trade-offs between yield and nutrient content.

Is this then an “Occam’s razor” explanation for the observation that rising [CO_2_] is resulting in a ubiquitous dilution in protein and minerals? Such a response would be consistent with any [CO_2_]-stimulation effect on photosynthesis and growth. 

**Figure 1:**
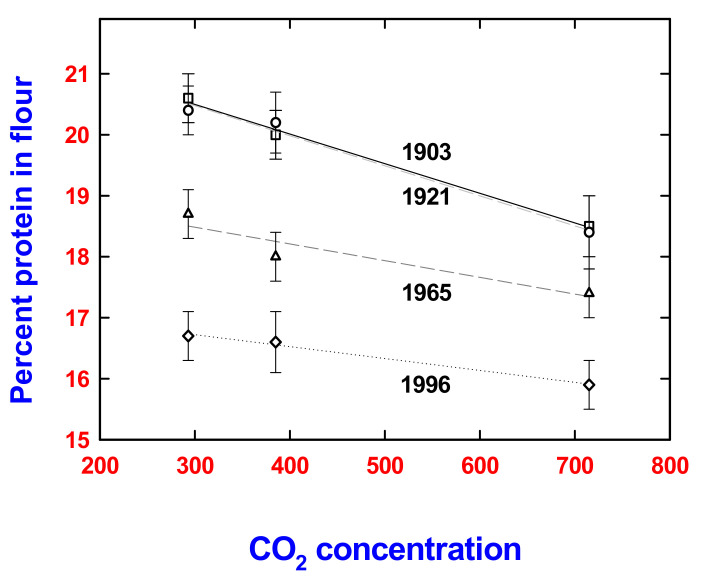
Selected changes in average grain quality (percent flour protein) showing ±SE as a function of CO_2_ concentration for the 20th century for different spring wheat lines. Data are redrawn from [13].

Yet, at present there is limited evidence for a simple dilution hypothesis. For example, a study of spring wheat lines from the early to late 20th century did indicate that newer lines had less protein in flour; however, each cultivar also showed a decline in protein with increasing [CO_2_] from the early 20th century (Figure 1). As spring wheat does not outcross per se, this suggests that [CO_2_] is directly affecting protein concentration separate from breeding history [13].

A dilution hypothesis is not supported from numerous studies where the degree of nitrogen or protein dilution is independent of the relative stimulation of elevated [CO_2_] on grain yield. For example, in a meta-analysis of 11 field-grown wheat cultivars, in over eight countries, grain protein content decreases on average by 7% under elevated [CO_2_], but the extent of the decline was independent of N application rate [14].

Overall, the basis for the nutritional decline is still being investigated [15]. An alternative hypothesis to dilution may be that less demand for nutrients occurs under elevated [CO_2_] conditions, e.g., less Rubisco e.g., [16,17]; however, this has not been specifically examined in the context of whether less nutrient demand is proportional to the degree of [CO_2_]-induced stimulation with additional nitrogen e.g., [14]. Lower plant nitrogen concentrations have also been suggested in response to decreased photorespiration at elevated [CO_2_], as reductions in photorespiration could inhibit shoot nitrate assimilation [18]; however, in field systems, ammonia is quickly converted to nitrate (days) [19] and such an explanation would not account for a decline in other elemental nutrients (e.g., Fe, Zn) [7]. In the long-term, reduced [CO_2_] induced declines in nutritional composition and could also result in a reduction in decomposition rates and reduced availability of critical nutritional components such as nitrogen [20]. However, such an explanation would not account for reductions in nitrogen concentration in well-fertilized annual crops or single season experimental results [14].

One factor of interest may be the indirect effect of rising [CO_2_] on stomatal aperture. As [CO_2_] increases, leaf stomata close and transpirational water flux is reduced. In turn, elemental uptake associated with mass flow is reduced [21]. This is consistent with observations that nutrients that were transported towards the roots primarily through transpiration-driven mass flow exhibited larger concentration decreases at elevated [CO_2_], compared to nutrients that mostly relied on diffusion, consistent with this hypothesis [15]. However, additional data, especially from C_4_ crops, is needed to confirm such as response.

## 3. Temperature, CO_2_ and Nutritional Balance

Carbon dioxide is not just the source of carbon for photosynthesis and growth, but a longwave-radiation trapping gas, with consequences for surface temperatures. Concurrent changes in both parameters are likely. Given this, how will simultaneous increases in [CO_2_] and temperature alter plant nutrition?

Overall, less experimental data is available that has examined both parameters relative to protein and mineral concentrations. For a soybean cultivar grown in situ at warmer temperatures, elevated [CO_2_] reduced Fe and Zn, but high temperature and elevated [CO_2_] showed no reductions relative to the control [22]. Wang et al. [23] reported that warming could only partially negate the impact of elevated [CO_2_] on grain protein concentration at the expense of grain yield, not fully offsetting the negative effects of climate change on crop production. Recently, Wei et al. [24] showed no compensatory effect of elevated temperature and CO_2_ concentration relative to elevated [CO_2_] per se; rather, some amino acid profiles declined further with [CO_2_] and temperature increased relative to the control (Table 1). Other field-based experiments have shown a decline in protein, with increasing [CO_2_] and no beneficial effect of warming temperatures, e.g., rice [25] and forage grasses [26].

There is an obvious need to interpret any [CO_2_]-related declines in protein and mineral nutrition relative to other physical changes associated with anthropogenic climate change, including temperature, precipitation and extreme events. However, while data are limited, there is no consistent evidence that warmer temperatures will negate any [CO_2_]-induced changes in protein and/or mineral nutrition.

## 4. Assessment: Human Nutrition

With respect to staple crops, a meta-analysis of 228 observations on barley, potato, rice and wheat reported reductions in protein concentrations that ranged from ca −10 to −15% [6]. A more extensive meta-analysis of 7761 pairs of observations over 130 species and cultivars reported an average 8% decline in mineral concentrations, excepting Mn [7]. A meta-analysis of vegetable responses (57 articles and 1015 observations) reported declines in the concentrations of protein, nitrate, magnesium, iron and zinc by 9.5%, 18%, 9.2%, 16.0% and 9.4%, respectively [9].

In assessing these data for trends, there are some generalizations. First, while protein and mineral concentrations decline, carbon-based compounds may increase. This increase is consistent across a wide range of crop species [7]. For example, for the meta-analysis of vegetables, elevated [CO_2_] increased the concentrations of fructose, glucose and total soluble sugar by 14.2%, 13.2% and 17.5%, respectively [9]. Secondly, the decline in protein and minerals does not occur (or to a smaller extent) in leguminous plants such as soybean and peanut when grown at elevated [CO_2_] [7]. Finally, there is agreement that C_4_ grasses will show less or no decline in quality with elevated [CO_2_] [27]. In context, however, among the top-10 production food crops, soybean is the only legume, and corn and sorghum the only C_4_ species [28]. Consequently, any qualitative changes related to rising [CO_2_] will have substantial consequences for human nutrition and public health.

These consequences can be evaluated with respect to three nutritional parameters that show a ubiquitous decline in response to elevated [CO_2_] in studies and meta-analyses: protein, iron (Fe) and zinc (Zn). Plants provide the bulk of global nutrients [29]: 63% of dietary protein, 81% of the iron and 68% of the zinc. As stressed by Smith et al. [30], reducing the nutritional density of plant sources could significantly affect the incidence and severity of nutritional deficiency on a global basis, at a time when over two billion people are already estimated to be nutritionally wanting.

Smith and Myers [31] did a global evaluation of elevated [CO_2_] consequences on dietary intake or iron, zinc and protein for 151 countries, taking into account age and sex and assuming constant diets while excluding other climatic impacts that could affect food production. Assuming a 2050 population and moderate [CO_2_] projections (550 ppm CO_2_ by mid-century), they estimated that an additional 175 million people will become zinc- deficient and an additional 122 million will become protein-deficient. For iron, they estimate that 1.4 billion women of childbearing age and children under 5 for countries with at least 20% anemia prevalence would lose ~4% of dietary iron.

Beach et al. [32] used projections of mid-century (550 ppm CO_2_) combined with modelled future food supply results to estimate nutrient availability by country. They estimate that projected increases in atmospheric [CO_2_] and nutritional declines in combination with [CO_2_] fertilization effects and climate influence on productivity will decrease the global availability of nutrients by 19.5% for protein, 13.6% for iron and 14.6% for zinc, relative to anticipated technology and market gains by 2050 [32]. Overall, the countries that currently suffer from high levels of nutrient deficiency would continue to be disproportionally affected.

The global health impacts of [CO_2_]-induced reductions in nutritional integrity are considerable, and there is additional detail currently available as to the severity and scope of these effects [33,34,35]. Overall, there is merited concern that hidden hunger, a recognition of chronic dietary deficiencies, will be exacerbated by increasing [CO_2_]. Children and pregnant women are especially vulnerable to nutritional deficits [36]. In addition, insufficient protein intake, consistent with micronutrient deficiencies, can restrict growth and tissue repair and result in low birthweight, wasting, stunting and other health issues associated with 2.2 million annual deaths in children less than 5 years of age. Zinc deficiency is estimated to cause approximately 100,000 deaths per year in children less than 5 years of age. The global burden of disease associated with iron deficiency has been estimated at ca 200,000 deaths and 45 million disability-adjusted life-years annually [37].

## 5. Assessment: Global Food Webs

There is warranted concern regarding the evidence for the ubiquitous effect of increasing [CO_2_] on nutritional quality, human diets and health impacts. Yet, from a systematic perspective, human diets are dependent on a small fraction of available food sources, i.e., about 75% of human food is derived from 12 plant and 5 animal species [38].

Given the evidence that the effect of rising CO_2_ on diminishing plant nutrition may be global in scope, it is imperative to also understand how such an ongoing change would impact all of the food web. Plants, from an ecological system’s viewpoint, are the planet’s primary producers; consequently, any environmental aspect that affects their function ([CO_2_] induced declines in protein and minerals), will have comprehensive consequences.

Such impacts could be indirect, occurring in response to less foliar nitrogen/protein and slower decomposition, with limited nutrient turnover at the ecosystem level [20,39]. However, such an indirect effect would be counter to observations that the supply of nitrogen has been increasing since the mid-1900s in response to anthropogenic deposition of reactive N from fertilizer production and fossil fuel combustion [40]. Interestingly, a seminal study by McLaughlin et al. [41] examined the composition of stable carbon (C) and N isotopes in leaf tissue from 545 herbarium specimens of 24 vascular plant species collected in Kansas, USA from 1876 to 2008, and found that N availability had declined in these grasslands despite decades of N deposition. Similarly, herbarium samples of Canadian goldenrod showed an approximate 30% decline in N over the same period [42]. These data are consistent with a progressive nitrogen limitation hypothesis and potential increases in ecosystem N storage in response to increased atmospheric [CO_2_] [41]. A direct effect is also possible. As noted previously [14,15], any decline in N due to elevated [CO_2_] was observed as independent of N application rate, i.e., there could be a direct reduction in N concentration separate from an anthropogenic deposition of N.

As with human food sources, direct or indirect effects of rising [CO_2_] on nutritional quality may also result in physiological consequences. Such outcomes would be particularly relevant in plant–insect interactions. Such interactions, or mutualisms, are a fundamental cornerstone in the generation and maintenance of life on earth, including pollination, protection and seed dispersal [43]. However, if recent and projected [CO_2_] affects nutritional concentrations, e.g., pollen [42], what are the implications for global food webs?

One potential outcome is highlighted by Welti et al. [44]. In this seminal study, they examined long-term datasets of grasshopper abundance in the Kansas prairie relative to climate and seasonal elemental content. Consistent with a nutrient dilution hypothesis, grass biomass doubled, but foliar concentrations of N, P, K and Na declined. The decline accounted for 25% of the variation in grasshopper populations (the dominant herbivore in the system) over a two-decade period. This suggested that a warmer, more CO_2_-enriched environment could, in addition to other environmental constraints, such as habitat loss and chemical pollution, lead to a decline in insect herbivores.

Insect populations, given their diversity and size, are difficult to monitor, but overall, any wide-spread nutritional decline would have substantial consequences for insect fecundity and demographics, as well as for animals higher up in the food web. Such consequences would also be evident for animals that consume plant material directly, including koalas and pandas.

## 6. Fundamental Unknowns

### 6.1. Mechanism

Although there is consensus that projected increases in [CO_2_] will adversely affect protein (nitrogen) and other elemental minerals (Fe, Zn), the mechanistic basis for the response is still unclear. A dilution process commensurate with [CO_2_] stimulation of growth is often cited as the basis for the observed reductions; however, this seems unlikely as nutrient concentrations are still decreased when growth is unaffected by elevated [CO_2_] [15]. Indirect effects of reduced transpirational flow from [CO_2_]-induced changes in stomatal closure could also account for nutritional reductions, but similar reductions in C_4_ species should also be evident and are not consistently observed [27]. Additional data specific to other key nutrients (e.g., iodine) that have not been quantified are also needed [7,35]. Finally, research into decomposition of nutrient deficient plant materials and soil effects related to storage and nutrient transfer are essential to understand long-term mineral availability.

### 6.2. Other Elements

As stressed by Ebi et al. [35], although every chemical element necessary for plants is also necessary for humans, the opposite is not true. Elements, including sodium (Na), iodine (I), lithium (Li), selenium (Se), chromium (Cr) are non-essential to plants but necessary to humans [45]. Conversely, other elements, most notably arsenic and lead, can be present and benign in plants, but are known toxins to humans [46]. Yet, at present, our knowledge regarding the role of rising [CO_2_], or [CO_2_] and climate, relative to changes in the concentrations of these elements in plant sources, is extremely limited [47].

### 6.3. Interactions with Anthropogenic Parameters

The ongoing increase in [CO_2_] is not separate from other anthropogenic changes, notably temporal and geographic shifts in temperature and precipitation, as well as projected changes in extreme weather events. As evidenced by [CO_2_] by temperature interactions relative to [CO_2_] effects on nutrition [22], additional evidence is essential to ascertain the cohesion of [CO_2_] effects on nutritional integrity with concurrent physical changes (e.g, drought). Such data would also assist to distinguish and understand physiological mechanisms related to [CO_2_] and nutritional use and the scope of [CO_2_]-induced changes in nutritional plant profiles.

### 6.4. Global Food Webs

There has been an understandable focus on the role of rising [CO_2_] specific to human nutrition. However, the broad range of [CO_2_]-induced declines in nutrition observed for a number of plant taxa warrants much more research into the consequences for global food webs. For example, studies from Germany and Puerto Rico suggest significant, rapid declines in insect populations [48], and while a number of anthropogenic influences are of interest, insects, like plants, are essential to every terrestrial food web, and any link to [CO_2_]-induced changes in plant nutritional quality deserves immediate entomological consideration. Overall, there is a clear and pressing need to understand the direct role of rising [CO_2_] on ecological processes, with a merited focus on biodiversity.

### 6.5. Plant Chemistry

Given the general shift in stoichiometry with additional [CO_2_], from a carbon rich, nutrient poor composition, it is compelling to ascertain if such shifts are also altering secondary plant chemistry. From a human perspective, elevated [CO_2_] has been associated with declines in carotenoid concentration [49], as well as B vitamins, nicotine, scopolamine, atropine and opioids [50,51,52]. From an ecological, food-web perspective, rising [CO_2_] can compromise plant chemical defenses against invasive insects [53]. The basis for these changes is unclear, and it has been suggested that the increase in carbon relative to other elements is reflected in secondary chemistry as a resource-use hypothesis; however, such a response is not consistently evident [54].

## 7. Research Imperatives

Data relative to genetic, physiological and environmental factors associated with mineral uptake, transport and utilization are necessary to understand the mechanistic role of elevated [CO_2_] [55]. Data on additional minerals (e.g., I, Li) in response to [CO_2_] would help to define these factors and to determine commonalities. Such a broader study of crop species should, if possible, be undertaken using a wider range of growing conditions likely to be encountered with anthropogenic climate change (e.g [CO_2_] and drought, [CO_2_] and temperature). Increasing our understanding of mechanistic pathways that link [CO_2_] (and climate change) to nutrition is a necessary step to develop effective interventions to ensure global food security.

The need to compensate for [CO_2_] reductions in crop nutrition is an obvious research priority. Such compensation can be at the genetic or the management level. Genetically, it is interesting to note the range of [CO_2_]-induced changes in nutritional concentration. For example, Zhu et al. [50] demonstrated a significant [CO_2_] by Zn interaction in rice, suggesting inherent genetic variability in maintaining Zn level as [CO_2_] increases. Potentially, high throughput screening [56] could provide insight into genetic traits associated with nutritional maintenance in response to rising [CO_2_]; however, at present, the use of such technologies to ascertain intra-specific nutritional variation among multiple crop lines has not been attempted. However, such information would also be invaluable for genetic transformation and GMO development for lines that would maintain or increase elemental concentration as [CO_2_] increases. Biofortification efforts can also be supplemented with other agronomic techniques, especially soil management, e.g., the addition of Zn fertilizer in rice fields. While these are laudable goals, it should also be recognized that for agronomic production in wealthy countries, mechanization and uniformity are preferred, whereas nutritional or qualitative variation is, in general, ignored.

There is also a potential benefit from genetic and agronomic management. Increased consumer awareness and selection of nutritional vegetables and fruit from producers who employ practices or genetics that reduce greenhouse gas (GHG) emissions is one incentive for diet diversity. Such an incentive would serve as “double-duty” in reducing agricultural contributions to anthropogenic warming. Policy incentives could also encourage producers to grow more diverse foods, or food companies to create more nutritionally- enhanced products while limiting GHGs [33].

While the emphasis on human food sources is understandable, research to ascertain and document the extent of [CO_2_]-induced changes in the nutritional integrity of global food webs is critical. It is not an exaggeration to suggest that these changes could affect all animal systems globally. Experimental data with notable exception [44] is lacking; yet, there are resources, such as herbariums, that could provide estimates of recent temporal increases in atmospheric [CO_2_] (+34% since 1950) relative to protein or micronutrient concentrations for a wide range of wild plant species [42].

There is, given the complexity of plant physiological responses, a need to improve current models of nutritional integrity and nutritional flow in order to elucidate the magnitude and direction of ongoing increases in [CO_2_], both in isolation and also as an integral response to climatic change. However, with important exceptions [57], such models are rare. Mechanistic, process-based models are needed for quantifying complete nutrient dynamics and stoichiometry, especially regarding human nutrition [35]. Transdisciplinary research, including collaboration and cooperation between ecologists, plant physiologists, dieticians, producers and economists is necessary in that regard. In addition, there is a need to include a wider array of specialists, including sociologists, public health experts and policy makers.

## 8. Final Thoughts

Popular images of climate change include starving polar bears, rising sea levels and super-charged hurricanes. All are instances of justified concerns regarding anthropogenic climate change. However, other impacts, as exemplified here by the extensive evidence indicating a wide-spread decline in nutritional concentration of numerous plant species with rising [CO_2_], will also have profound and wide-ranging effects on numerous aspects of global biology, not least of which is human nutrition. These effects are not trivial. Weyant et al. [58] estimated that CO_2_-induced reductions in micronutrients such as zinc and iron in crops could result in an additional 125.8 million disability-adjusted life years (DALYs) globally, with South-East Asian and sub-Saharan African countries most affected. Zhu et al. [50] estimated 600 million people at risk past mid-century are in the highest rice-consuming countries in Asia with the lowest overall gross domestic product per capita. Beach et al. [32] projected a 2.4% to 4.3% penalty on expected gains in global availability of protein, iron and zinc by mid-century because of technology change, market responses and the fertilization effects of [CO_2_] on yield. It is clear that understanding these effects is fundamental and crucial to estimate the consequences not only for public health, but also for the transfer and adequate functioning of the global food web.

It is puzzling and perplexing that in looking at available resources to both broaden the scope of our understanding and assess the global outcomes of [CO_2_] and nutrition, there is a profound lack of academic support and funding. No programs regarding [CO_2_] and human nutrition currently exist at the National Institute of Health (NIH) and no RFAs at the National Science Foundation (NSF) exist to examine the role of [CO_2_] on nutrition as part of the global food web; yet, these are agencies that, ostensibly, are designed to address significant scientific issues, in the public’s interest. As Howard Frumkin wrote in Scientific American [59], at present the total research funding on climate change for all health consequences is approximately nine million dollars, or 0.02% of the NIH’s 40-billion-dollar budget.

We can and we must do better, not only in terms of [CO_2_], plant and nutritional consequences, but in our ability to address the problem of anthropogenic climate change. If there is an unseen benefit to the current pandemic, ignoring public health consequences that can affect millions of lives is no longer an option. The evidence is here. Action is needed.

## Figures and Tables

**Table 1:** Changes in essential amino acid composition (mg g^−1^) in rice grown at ambient (a) and elevated CO_2_ and temperature (e), aCaT, ambient check; aCeT, elevated temperature (+1.5 °C), eCaT (+200 ppm CO_2_) and eCeT. Letters indicate significant differences. Data are from [24].

Amino Acid	aCaT	aCeT	eCaT	eCeT	CO_2_	Temp.
Histidine	1.62 a	1.61 a	1.47 ab	1.44 b	0.012	n.s
Isoleucine	2.39 a	2.38 a	2.14 a	2.14 a	n.s	n.s
Leucine	5.44 a	5.38 a	4.91 ab	4.81 b	0.018	n.s.
Lysine	2.45 a	2.49 a	2.27 bc	2.22 c	0.013	n.s.
Methionine	1.39 a	1.21 b	1.20 b	1.12 c	0.001	0.002
Phenylalanine	3.61 a	3.59 a	3.28 a	3.22 a	n.s.	n.s.
Threonine	2.66 a	2.67 a	2.46 ab	2.42 b	0.014	n.s.
Valine	3.99 a	3.97 a	3.60 a	3.59 a	n.s.	n.s.
TOTAL	23.55 a	23.31 a	21.34 ab	20.89 b	0.013	n.s.

## Data Availability

Data are available from the original sources as listed in References.
